# Impact of SMS and peer navigation on retention in HIV care among adults in South Africa: results of a three‐arm cluster randomized controlled trial

**DOI:** 10.1002/jia2.25774

**Published:** 2021-08-25

**Authors:** Wayne T Steward, Emily Agnew, Julia de Kadt, Mary Jane Ratlhagana, Jeri Sumitani, Hailey J Gilmore, Jessica Grignon, Starley B Shade, John Tumbo, Scott Barnhart, Sheri A Lippman

**Affiliations:** ^1^ Division of Prevention Science Department of Medicine University of California San Francisco CA USA; ^2^ International Training and Education Center for Health (I‐TECH) Pretoria Republic of South Africa; ^3^ Department of Global Health University of Washington Seattle WA USA; ^4^ International Training and Education Center for Health (I‐TECH) South Africa Pretoria Republic of South Africa; ^5^ Institute for Global Health Sciences Department of Epidemiology and Biostatistics University of California San Francisco CA USA; ^6^ Department of Family Medicine and Primary Health Care Sefako Makgatho Health Sciences University Pretoria Republic of South Africa

**Keywords:** retention in care, text messaging, peer navigation, South Africa

## Abstract

**Introduction:**

Few interventions have demonstrated improved retention in care for people living with HIV (PLHIV) in sub‐Saharan Africa. We tested the efficacy of two personal support interventions – one using text messaging (SMS‐only) and the second pairing SMS with peer navigation (SMS+PN) – to improve HIV care retention over one year.

**Methods:**

In a cluster randomized control trial (NCT# 02417233) in North West Province, South Africa, we randomized 17 government clinics to three conditions: SMS‐only (6), SMS+PN (7) or standard of care (SOC; 4). Participants at SMS‐only clinics received appointment reminders, biweekly healthy living messages and twice monthly SMS check‐ins. Participants at SMS+PN clinics received SMS appointment reminders and healthy living messages and spoke at least twice monthly with peer navigators (PLHIV receiving care) to address barriers to care. Outcomes were collected through biweekly clinical record extraction and surveys at baseline, six and 12 months. Retention in HIV care over one year was defined as clinic visits every three months for participants on antiretroviral therapy (ART) and CD4 screening every six months for pre‐ART participants. We used generalized estimating equations, adjusting for clustering by clinic, to test for differences across conditions.

**Results:**

Between October 2014 and April 2015, we enrolled 752 adult clients recently diagnosed with HIV (SOC: 167; SMS‐only: 289; SMS+PN: 296). Individuals in the SMS+PN arm had approximately two more clinic visits over a year than those in other arms (*p* < 0.01) and were more likely to be retained in care over one year than those in SOC clinics (54% vs. 38%; OR: 1.77, CI: 1.02, 3.10). Differences between SMS+PN and SOC conditions remained significant when restricting analyses to the 628 participants on ART (61% vs. 45% retained; OR: 1.78, CI: 1.08, 2.93). The SMS‐only intervention did not improve retention relative to SOC (40% vs. 38%, OR: 1.12, CI: 0.63, 1.98).

**Conclusions:**

A combination of SMS appointment reminders with personalized, peer‐delivered support proved effective at enhancing retention in HIV care over one year. While some clients may only require appointment reminders, the SMS+PN approach offers increased flexibility and tailored, one‐on‐one support for patients struggling with more substantive challenges.

## 1 Introduction

The preventive benefits of antiretroviral therapy (ART) are well established [1], but challenges remain with HIV care uptake. Recent national data from South Africa indicate that only 52% of people living with HIV (PLHIV) aged 15 or older are virally suppressed [2]. This comes despite improved medication access through policies for Universal Test & Treat (UTT), Same‐Day Initiation (SDI) of ART [3] and decentralized medication initiation and monitoring [4]. Although UTT has been associated with a 30% reduction in loss‐to‐follow‐up, nearly 40% of HIV patients continue to have suboptimal retention [3]. To further address barriers to care, countries are deploying personal support strategies, including short message service (SMS or text message) reminders [5] and in‐person support (e.g. from community‐based healthcare workers) [6].

Research on SMS communications shows that they can enhance HIV care outcomes. A systematic review of digital innovations found that SMS reminders improved adherence and clinic attendance [7], while another noted that African females had higher odds of returning to HIV care when receiving mobile phone reminders [8]. However, SMS impact on HIV retention outcomes has been mixed, with studies in Malawi and Kenya failing to find improvements in retention after deploying SMS interventions, [9, 10] and a trial in Mozambique noting improved retention among urban, but not rural, patients [11]. The wide accessibility of SMS makes it an important technology to consider, even as smartphones and associated communication apps become more available. Recent data found that 51% of South Africans used smartphones but 40% still had basic mobile phones, and that smartphone users were even more likely than basic phone user to send SMS (91% vs. 66%) [12].

In addition to SMS and other messaging interventions, there is evidence that personal support through face‐to‐face interactions can improve HIV care continuum outcomes. South African research has shown that adherence clubs, generally facilitated by lay healthcare workers, improve retention, with 90% of eligible patients still in care and virally suppressed (≤400 copies/mL) 12 to 24 months after initiating treatment [13, 14]. Other strategies include home‐based care services [15], identification of a “treatment supporter” [16] and health system navigators to promote linkage to care [17]. Peer‐based interventions have been shown in some studies to improve linkage to care and adherence while reducing healthcare system burdens [18].

We implemented a three‐arm cluster randomized trial in public primary health clinics and community health centres in North West Province, South Africa to examine the efficacy of two personal support interventions [19] at improving retention in HIV care. One used only automated SMS communications to deliver support (SMS‐only). The second paired SMS messages with peer navigation (SMS+PN). We compared both to standard of care (SOC) [19] using a clustered trial design, randomizing by clinic, to reduce the risks of cross‐arm contamination and ensure adherence to a uniform protocol at each clinic. We hypothesized that clients in both SMS‐only and SMS+PN clinics would have improved retention in HIV care over one year as compared to clients in SOC clinics.

## 2 Methods

### 2.1 Study setting

The cluster randomized controlled trial (CRCT) was conducted in the North West Province where HIV prevalence was high [20]: 13.3% among the general population and 28.2% in antenatal services at study launch [21]. The study was conducted in two sub‐districts, Moses Kotane and Rustenburg, of Bojanala Platinum District, where 37% of the provincial population resides and HIV prevalence is 31% in antenatal clinics [21]. Clinical sites included five community health centres and 13 primary health clinics. Both facility types offer HIV testing and treatment; community health centres also usually provide 24‐hour maternity and emergency services.

### 2.2 Randomization

**Figure 1:**
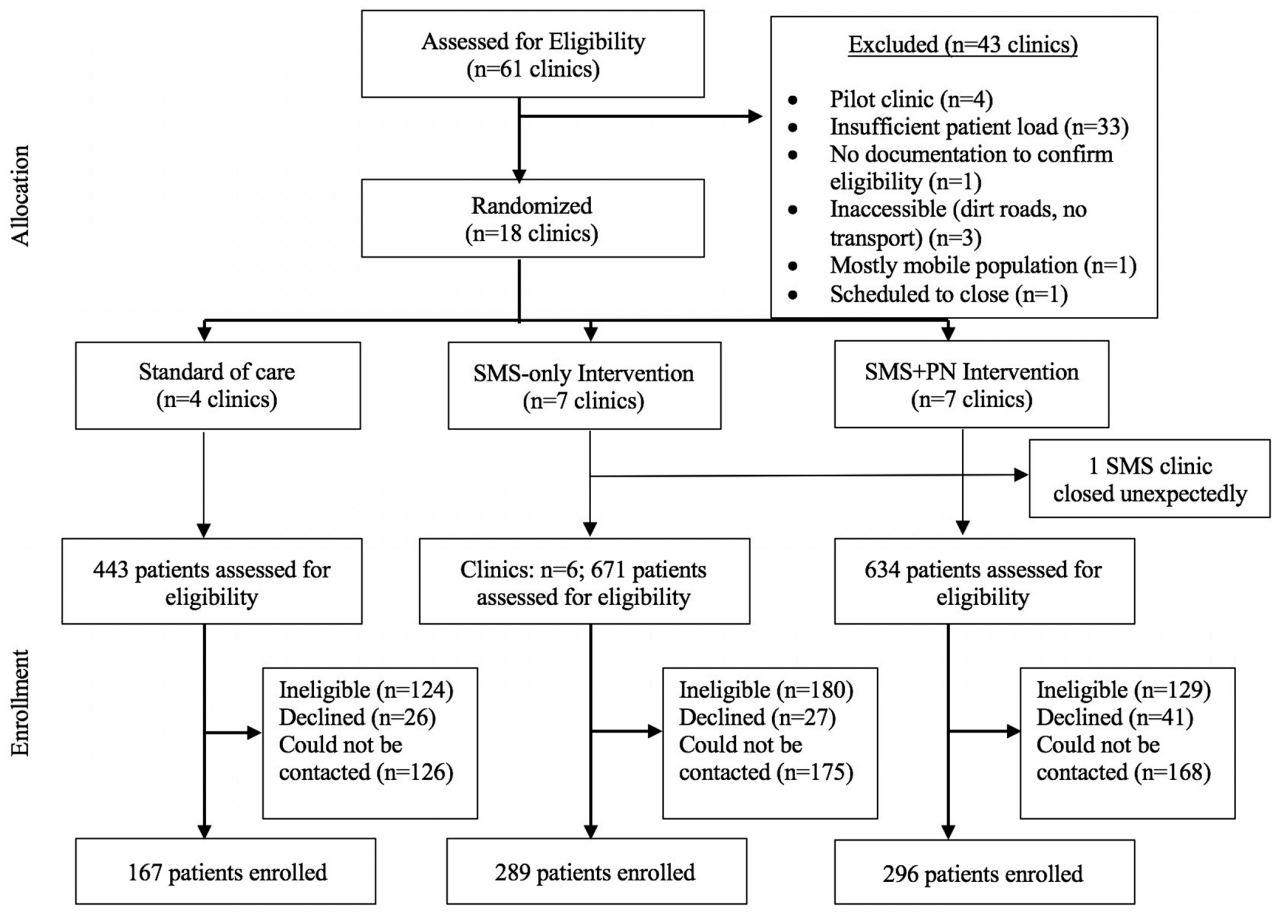
Trial profile.

Eighteen sites were selected from 61 facilities providing ART. A chosen site could not be one of four that piloted the interventions [22]; required a catchment area of ≥6000 inhabitants and needed adequate patient load to ensure recruitment targets (assessed as adding ≥40 new patients to HIV care registers from January through April, 2014). Among 24 sites meeting criteria, we excluded one primarily serving a mobile mining workforce and unlikely to retain patients; one not using patient registers (making eligibility criteria difficult to determine); one scheduled to close and three distant from study offices, complicating access and supervision. The remaining 18 facilities were randomized: seven to each intervention and four to SOC (Figure 1). As described previously [19], we used balanced (restricted) randomization based on indicators of patient load and clinic functionality, stratified by sub‐district [23].

Participant eligibility criteria included presenting at a study clinic, HIV diagnosis within the past year, being ≥18 years old, access to a mobile phone (94% of residents in North West Province had cell phone access in 2014 [24, 25]), and willingness to receive automated SMS with HIV‐related content. At trial initiation, South Africa had not implemented UTT. Therefore, participants could be receiving ART or pre‐ART services. Clinic staff assisted with recruiting patients presenting for HIV testing or initial CD4+ test results and by contacting a systematic sample of patients listed in HIV care registers in the past year. Clinic staff elicited permission for contact by study staff, who proceeded with enrolling eligible participants [19].

### 2.3 Procedures

The SMS‐only intervention used an automated messaging system (CommCare Connect, Dimagi, Inc., Boston, MA, USA) to deliver three kinds of SMS. First, reminders were sent prior to clinic appointments, and every two weeks after missed appointments until a participant returned to care or three months elapsed. Second, brief behavioural messages were sent biweekly to encourage engagement in care, adherence, prevention (e.g. disclosure to sexual partners) and healthy living (e.g. reduced alcohol use). Behavioural messages utilized both English and Setswana. They were scripted by the study team, piloted to ensure clarity and sent in a pre‐determined order [19]. Third, SMS check‐in messages were sent biweekly, asking participants to reply, free of charge using a numeric code, to indicate if they were experiencing challenges. Participants reporting difficulties or not responding were contacted to understand the nature of any problems and triage the matter to medical personnel at the participant’s clinic.

The SMS+PN intervention used the same automated system to send SMS appointment reminders and biweekly behavioural messages. Additional support was provided by peer navigators, who were PLHIV receiving care at a local facility [19, 22]. Navigators met each client in‐person at least once per month and had one phone or SMS check‐in mid‐month. Additional contacts were encouraged. Navigators worked with clients to identify and address barriers to care.

Participants at SOC clinics did not receive services beyond standard care. Personnel at facilities in all trial arms received training in proper completion of patient clinical forms to ensure consistent data quality. Monitoring and evaluation assistants were stationed at clinics to extract patient chart data, conduct surveys and provide support to clinics.

At enrolment, participants provided contact information and written informed consent. They responded to a survey, which was repeated at six and 12 months. Participants received 50 Rand (US $4.52 at trial launch) of cellular airtime at each survey wave. Trial procedures were conducted in English and Setswana. Survey questions captured sociodemographic characteristics, alcohol consumption and distance to the nearest facility [19]. Data on clinical visits, medication dispensed and laboratory results were extracted biweekly from clinic records.

### 2.4 Outcomes

Primary exposure was classified as clinic randomization arm: SMS‐only, SMS+PN or SOC. The trial outcome, retention in care, was measured at the individual level and defined by ART status. During the first two months of accrual, individuals were eligible for ART if they had CD4 cell count ≤350, were pregnant, and/or had TB co‐infection. Eligibility was expanded to CD4 ≤500 in January 2015 [26]. 128 people (17.0%) were recruited prior to the change. Study clinics routinely dispensed up to a 90‐day medication supply during patient visits. Over a 90‐day period, a person could miss ≤4 days of medication and have enough pills to meet the 95% adherence criterion established in South African guidelines [26]. Therefore, retention for ART patients was defined as having a care appointment at least once every 94 days. For pre‐ART patients, retention was defined as returning within two months of required semiannual CD4 monitoring visits [26]. For participants first prescribed ART during study follow‐up, retention was defined by pre‐ART standards up to ART enrolment, and by ART standards for the remainder of the person’s follow‐up.

### 2.5 Statistical analysis

We used an intent‐to‐treat (ITT) analytic approach. The trial was powered to detect a 20% to 23% difference in retention in care after 12 months between each intervention arm and the SOC arm, assuming 18 clusters with a minimum of 32 participants per cluster, a two‐sided alpha of 0.05, and a coefficient of variation of 0.15 to 0.20 (intra‐cluster correlation [ICC] of 0.0225 to 0.04). The trial was not powered to examine differences between intervention arms. Primary analyses were conducted in Stata version 14 (StataCorp, College Station, TX, USA); sensitivity analyses were conducted in R (R Foundation for Statistical Computing) using the “tmle” package.

We compared participant demographic characteristics and clinic attendance within each trial arm using measures of central tendency and cross‐tabular frequencies adjusted for clustered data. We examined retention in care by intervention arm using generalized estimating equations (GEE), with a logit link and assuming an exchangeable correlation structure. Analyses included the full cohort, stratified by sex and ART status. We utilized robust standard errors which are unbiased in a setting where the coefficient of variation is less than 0.20 [27]. We adjusted models to account for clustering by clinic, as well as by covariates associated with the outcome.

Sensitivity analyses to further adjust for potential confounding [28] employed targeted maximum likelihood estimation (TMLE) [29]. This approach allowed us to examine if odds ratio estimates changed when using machine learning to derive the best causal inference model. We did not use TMLE for primary analyses because its machine learning process adds to the estimate variability (widens the confidence intervals) [29].

### 2.6 Role of the funding source

The US Health Resources and Services Administration provided funding as part of a grant to improve HIV clinical services and prevention. The funder had no involvement in research instrument and protocol design, data collection, analysis and interpretation, writing this report or the decision to submit for publication.

## 3 RESULTS

Overall, 1748 patients across 17 clinics were approached by staff or selected from patient registers for potential enrolment. We were unable to reach 469 (27%) of those selected from registers. Of the 1279 contacted, 846 (66%) met eligibility criteria. Among those eligible, 752 (89%) agreed to participate (SOC: 167; SMS‐only: 289; SMS+PN: 296) and 94 (11%) declined (Figure 1). The 18th recruitment clinic, randomized to the SMS‐only intervention, was dropped because it closed unexpectedly for construction prior to trial launch and did not reopen. Enrolment at other SMS‐only sites was increased to offset the loss.

Approximately 61% of participants were females, just over half were younger than 35%, 59% were married or in a relationship, 81% had at least a secondary school education, 32% lived below the food poverty line (defined as <ZAR 400/month [30]) and 53% lived within 30 minutes of the closest clinic (Table 1). Nearly all (98.6%) owned a mobile phone, but 85% reported not always having sufficient airtime or data. At enrolment, 479 participants (64%) were considered pre‐ART, but only 16% (n = 124) remained pre‐ART at last study follow‐up. In subsequent analyses, participants’ ART status is based on ART initiation as of last study assessment. The only difference in baseline participant characteristics across trial arms was the proportion of females pregnant at HIV diagnosis, which was higher among SOC participants (61% compared to 34% for SMS‐only and 31% for SMS+PN).

**Table 1:** Demographic characteristics of the participants

Characteristics	All trial arms	SOC	SMS‐only	SMS+PN	
	N = 752	n = 167	n = 289	n = 296	Chi‐squared
	n (%)	n (%)	n (%)	n (%)	*p*‐value
Sex					0.49
Male	292 (38.8)	70 (41.9)	116 (40.1)	106 (35.9)	
Female	460 (61.2)	97 (58.1)	173 (59.9)	190 (64.2)	
Age					0.84
18 to 24	113 (15.0)	32 (19.2)	38 (13.2)	43 (14.5)	
25 to 29	163 (21.7)	39 (23.4)	71 (25.6)	53 (17.9)	
30 to 34	130 (17.3)	29 (17.4)	52 (18.0)	49 (16.6)	
35 to 39	128 (17.0)	23 (13.8)	48 (16.6)	57 (19.3)	
40 to 49	140 (18.6)	29 (17.4)	54 (18.7)	57 (19.3)	
40 to 69	78 (10.4)	15 (9.0)	26 (9.0)	37 (12.5)	
Marital status					0.27
Single	305 (40.6)	82 (49.1)	87 (30.2)	136 (46.0)	
Married/in relationship	446 (59.4)	58 (59.2)	201 (69.8)	160 (54.1)	
Educational attainment					0.94
Primary or less	143 (19.0)	28 (16.8)	58 (20.1)	57 (19.3)	
Secondary or more	608 (81.0)	139 (83.2)	230 (79.9)	239 (80.7)	
Pregnant at diagnosis (females only)					0.02
No	282 (61.3)	38 (39.2)	114 (65.9)	130 (68.4)	
Yes	175 (38.0)	59 (60.8)	58 (33.5)	58 (30.5)	
Missing	3 (0.0)	0 (0.0)	1 (0.6)	2 (1.1)	
South African citizen or resident					0.98
No	110 (14.7)	25 (15.0)	44 (15.3)	41 (13.9)	
Yes	641 (85.4)	142 (85.0)	244 (84.7)	255 (86.2)	
Living below food poverty line (ZAR 400/month)					0.91
No	467 (68.2)	101 (68.2)	183 (69.9)	183 (65.6)	
Yes	218 (31.8)	47 (31.8)	79 (30.2)	92 (33.5)	
Harmful drinking^a^					0.11
No	659 (89.1)	145 (87.4)	266 (94.0)	248 (85.2)	
Yes	81 (11.0)	21 (12.7)	17 (6.0)	43 (14.8)	
Time to nearest facility					0.28
0 to 30 minutes	400 (53.2)	75 (80.2)	146 (50.7)	179 (60.5)	
31 to 60 minutes	313 (41.7)	87 (52.1)	130 (45.1)	96 (32.4)	
61 to 90 minutes	38 (5.1)	5 (3.0)	12 (4.2)	21 (7.1)	

^a^ Harmful drinking was assessed using the Alcohol Use Disorders Identification Test (AUDIT) [31, 32, 33].

Individuals in the SMS+PN arm had approximately two more clinic visits over the one‐year follow‐up period than those in the SOC or SMS‐only arms (Table 2). In analyses stratified by sex and ART status, similar patterns were observed among all subgroups, although differences failed to achieve statistical significance among pre‐ART females.

**Table 2:** Average number of HIV‐related clinic visits by participants during the one‐year follow‐up period

	All trial arms combined		SOC		SMS‐Only		SMS+PN		Difference across arms^a^	
	Mean	SD	Mean	SD	Mean	SD	Mean	SD	(*p*‐value)
All participants	6.32	4.05	5.52	3.90	5.43	3.67	7.65	4.14	0.001	
Males only	6.07	4.16	5.21	3.89	5.06	3.65	7.73	4.35	0.001	
Females only	6.49	3.98	5.74	3.90	5.68	3.67	7.61	4.03	0.001	
Pre‐ART participants only	1.09	2.15	0.36	0.62	0.60	1.30	2.24	3.09	0.003	
On ART participants only	7.36	3.50	6.56	3.42	6.56	3.07	8.52	3.60	0.001	
Pre‐ART males only	0.84	2.05	0.21	0.58	0.16	0.37	2.28	3.20	0.001	
Pre‐ART females only	1.30	2.22	0.50	0.65	0.97	1.65	2.22	3.07	0.104	
On ART males only	7.33	3.50	6.46	3.31	6.41	2.92	8.84	3.67	0.001	
On ART females only	7.37	3.51	6.63	3.51	6.66	3.18	8.35	3.56	0.001	

^a^ Kruskal–Wallis equality of means test to assess differences in mean visits by arm for each participant group.

To account for potential confounding, we examined associations between participant characteristics and retention. Participants were significantly more likely to be retained if they were female, older, single and living within 30 minutes of the nearest clinic (Table 3).

**Table 3:** Association of participant characteristics with retention

	Retained in care		
	OR	95% CI	
Sex			
Male	1.00		
Female	1.41	0.97	2.04
Age			
18 to 24	1.00		
25 to 29	1.24	0.80	1.92
30 to 34	1.18	0.67	2.09
35 to 39	1.77	1.04	3.01
40 to 49	2.11	1.17	3.78
50 to 69	2.49	1.51	4.11
Marital status			
Single	1.00		
Married/in relationship	0.63	0.44	0.88
Educational attainment			
Primary or less	1.00		
Secondary or more	1.18	0.85	1.64
Pregnancy at diagnosis (females only)			
No	1.00		
Yes	0.74	0.53	1.04
South African citizen/resident			
No	1.00		
Yes	1.61	0.92	2.82
Living below food poverty line (ZAR 400 per month)			
No	1.00		
Yes	1.09	0.81	1.47
Harmful drinking			
No	1.00		
Yes	1.02	0.64	1.65
Time to nearest facility			
0 to 30 minutes	1.00		
31 to 60 minutes	0.69	0.54	0.89
61 to 90 minutes	0.59	0.37	0.94

Table 4 presents the trial’s primary intention to treat analyses, adjusted for clustering by clinic and for age, marital status and time to the nearest facility. The ICC for retention in care at 12 months was 0.07 (95% CI: 0.01 to 0.12), similar to the estimate utilized for sample size calculations; the coefficient of variation was 0.040. Significantly more participants in SMS+PN clinics were retained in care than in SOC clinics (54% vs. 38%; OR: 1.77, CI: 1.02, 3.10). In stratified analyses (Table 4), this difference remained significant among those on ART (61% vs. 45% retained; OR: 1.78, CI: 1.08, 2.93). (Statistical comparisons were not possible for pre‐ART participants due to low numbers retained.) Differences were not significant between SMS+PN and SOC arms when analyses were stratified by sex (males: 50% vs. 36% retained, OR: 1.62, CI: 0.64, 4.09; females: 57% vs. 39% retained, OR: 1.75, CI: 0.96, 3.22). The SMS‐only intervention did not demonstrate significant effects relative to SOC, either in unstratified analyses (40% vs. 38% retained, OR: 1.12, CI: 0.63, 1.98) or when stratified by ART status (ART participants: 49% vs. 45% retained; OR: 1.15, CI: 0.69, 1.90) or sex (males: 34% vs. 36% retained; OR: 0.98, CI: 0.37, 2.60; females: 45% vs. 39% retained, OR: 1.15, CI: 0.62, 2.13).

**Table 4:** Retention in HIV care over 12 months across trial arms

Trial	N	Number	Percent	Odds	95% CI
Arm		Retained	Retained	Ratio	
All participants					
SOC	167	63	37.72	1.00	
SMS‐only	289	116	40.14	1.12	0.63 to 1.98
SMS+PN	296	161	54.39	1.77	1.02 to 3.10
Males only					
SOC	70	25	35.71	1.00	
SMS‐only	116	39	33.62	0.98	0.37 to 2.60
SMS+PN	106	53	50.00	1.62	0.64 to 4.09
Females only					
SOC	97	38	39.18	1.00	
SMS‐only	173	77	44.51	1.15	0.62 to 2.13
SMS+PN	190	108	56.84	1.75	0.96 to 3.22
Pre‐ART only					
SOC	28	0	0.00	^a^	
SMS‐only	55	2	3.64	^a^	
SMS+PN	41	5	12.20	^a^	
On ART only					
SOC	139	63	45.32	1.00	
SMS‐only	234	114	48.72	1.15	0.69 to 1.90
SMS+PN	255	156	61.18	1.78	1.08 to 2.93
Pre‐ART males only					
SOC	14	0	0.00	^a^	
SMS‐only	25	0	0.00	^a^	
SMS+PN	18	1	5.56	^a^	
Pre‐ART females only					
SOC	14	0	0.00	^a^	
SMS‐only	30	2	6.67	^a^	
SMS+PN	23	4	17.39	^a^	
On ART males only					
SOC	56	25	44.64	1.00	
SMS‐only	91	39	42.86	0.94	0.44 to 2.00
SMS+PN	88	52	59.09	1.65	0.77 to 3.53
On ART females only					
SOC	83	38	45.78	1.00	
SMS‐only	143	75	52.45	1.25	0.66 to 2.39
SMS+PN	167	104	62.28	1.75	0.93 to 3.31

Confidence intervals are adjusted for clustering and for covariates independently associated with retention: age, marital status and time to the nearest facility. ART, antiretroviral therapy; SMS+PN, intervention using SMS and peer navigation; SMS‐only, Intervention using only short message service (text messaging); SOC, Standard of Care.

^a^ There were no pre‐ART participants retained in the SOC arm and a total of seven retained in the intervention arms. As such, in stratified analyses, we were not able to calculate odds ratios to compare outcomes between SOC and intervention arms for pre‐ART participants.

In sensitivity analysis (see Table S1), TMLE‐derived odds ratio estimates remained near identical to those obtained in primary analyses. As expected [29], confidence intervals were wider.

## 4 Discussion

We found that an intervention combining SMS appointment reminders, behavioural messaging and support from peer navigators nearly doubled the odds of recently diagnosed PLHIV being retained in HIV care over one year, relative to standard of care. Importantly, the findings were driven by differences among participants on ART. Across study arms, those on ART were more likely to be retained than those who were pre‐ART, consistent with findings following UTT rollout [3]. Among participants on ART, retention was significantly higher when they had received the SMS+PN intervention. The results thus suggest that the SMS+PN intervention can be an important tool for maintaining continuity of treatment among patients in the UTT era, which is characterized by universal access, but also continued HIV stigma [34, 35] and retention challenges [2].

Our findings also indicate that an SMS‐only intervention was not successful at improving retention. This finding adds to the literature documenting mixed effects for SMS strategies [7, 8, 9, 10, 11]. The intervention’s focus primarily on appointment reminders may have been sufficient for addressing limited retention barriers (e.g. forgetfulness) but insufficient for complex stigma‐related barriers, such as fearing HIV status disclosure. In pilot findings, we learned that navigators were seen as sources of inspiration, which was helpful in addressing stigma‐related barriers [22]. The success of our SMS+PN intervention suggests that interventions incorporating SMS may work better when they enable flexible communications and allow participants to bond with a support person sending the messages. Personalized messaging, however, is not standard for most SMS interventions, which utilize structured communications [7, 8, 9, 10, 11, 36, 37]. In South Africa, for example, one of the most established SMS interventions is MomConnect, which utilizes twice‐weekly standard health education texts to promote uptake of maternal and child health services among pregnant females, including those living with HIV [38]. MomConnect has demonstrated feasibility, but effectiveness is not yet evaluated [39]. Expansion of the SMS‐only intervention approach to newer, more flexible platforms could allow for less restrictive messaging and potentially improve messaging‐based intervention efficacy.

Our findings have important implications given the current South African context. Recent retention outcomes have varied greatly by programme. At one end, ART clubs have high retention (90% retained up to 24 months post‐diagnosis) [13, 14], however, with eligibility restricted to patients who first reliably engage in care at clinics [40], findings are indicative of a population that may have fewer retention challenges. At the other end, studies have identified higher rates of loss‐to‐follow‐up among patients linked to ART on the day of diagnosis, with such patients being lost on average within 55 days [41]. This finding is likely due to same‐day initiation policies improving linkage outcomes most strongly among those with substantial engagement challenges, who are then quickly lost. The striking differences in outcomes between ART clubs and SDI highlight the value of differentiated care models that direct enhanced support to patients facing more intense retention challenges [42]. The provision of enhanced personal support through the SMS+PN intervention could have an important role in differentiated care. Peer and lay health worker interventions in sub‐Saharan African settings have typically shown success with shorter‐term outcomes, such as linkage to care [17, 18]. It is thus notable that the SMS+PN intervention was effective at improving retention over one year. Targeting SMS+PN services to the portion of HIV patients most in need might also facilitate bringing the intervention to scale. While the SMS+PN intervention is equipped to tackle more complex barriers, it requires greater personnel time and resources than the SMS‐only intervention. Key to intervention optimization will be ensuring that SMS and PN support are delivered to PLHIV specifically when they are facing more intense retention challenges, which is most often in the first few months of care.

We did not observe significant differences stratifying data by sex, but this may be due to limited statistical power. The intervention effect size for females was nearly identical to that seen in unstratified analyses whereas the effect size for males was smaller (1.62 vs. 1.77). Females were also more likely to be retained overall, aligning with findings [43, 44] suggesting that males are less likely to access care due to structural barriers [45, 46] and fear of HIV status disclosure [47]. Our SMS+PN intervention may need to be augmented with elements to bolster impact among males, for example tying navigation and SMS reminders to community‐based or home‐based ART distribution [46].

A few contextual changes have occurred since this trial. Data were collected prior to UTT [3], however, our results remain highly relevant. The SMS+PN intervention worked most successfully among those qualifying for ART. Furthermore, our prior pilot work suggested that many barriers to care addressed by navigators were stigma related [22]. Such concerns are likely to remain highly salient even among patients in the UTT era being offered ART earlier in their disease course [34, 35]. Our data also reflect the time before the COVID‐19 pandemic, which restricts in‐person contacts and necessitates new intervention delivery approaches. The SMS+PS programme could potentially be conducted virtually. We informally observed during our study that participants welcomed occasional remote support, as necessitated by circumstance (e.g. navigator phone call when participant was traveling). This offers suggestive evidence that virtual navigation might work, although differences between in‐person and fully virtual navigation would need to be thoroughly evaluated in a trial.

Limitations in this trial include the lack of a viral suppression outcome. Although national guidelines recommended viral load testing six months after initiating ART and annually thereafter [26], viral load was not conducted or reported consistently. Additionally, the technology component of our work was restricted to SMS, which has greater limitations than communication apps available through smartphones. The use of smartphones could offer more flexibility, allowing clients to pick a preferred messaging platform, albeit with the caveat that such apps would not serve the substantial number of people in South Africa who still use basic mobile phones [12, 48]. Importantly, the principal barriers to our participants’ mobile phone use – specifically, lack of connectivity and insufficient funds to purchase data – would be obstacles for any phone‐based messaging platform. Only 50% of rural residents, compared to 66% of urban residents, in North West Province have access to the Internet through a mobile phone [25]. Such challenges are recognized contributors to the digital divide in emerging economies [49]. Finally, while the trial was not powered to formally assess differences between the two intervention arms, findings imply that the SMS+PN intervention had a larger and more robust impact. Despite limitations, the rigor of the CRCT design, using clinical charts and continuous retention outcomes, adds to the current literature on the need for personal support programming in areas where retention falls short.

## 5 Conclusions

Enhancing retention in care is critical if South Africa is to achieve the goals of the national and UNAIDS targets [50] and ensure that the vast majority of PLHIV are virally suppressed. Our SMS+PN intervention offers a promising strategy to address gaps in the country’s HIV continuum of care [43]. Research is now needed to determine how this intervention can be scaled up in an efficient and cost‐effective manner to address retention regionally.

## Competing interests

The authors declare no conflict of interest.

## Authors' contributions

SL was the principal investigator and conceived of the study and study design with WS, SS, JT and SB. JD, MJR, JS, HG and JG trained field staff and supervised data collection. EA, JD and HG coordinated data management and quality control procedures. WS led the development of the manuscript; EA prepared and performed the analyses with SS; all other authors contributed to manuscript editing and review. All authors contributed to the intellectual content of the manuscript, to the development of the trial protocol and read and approved the final manuscript.

## Abbreviations

ART, antiretroviral therapy; CRCT, cluster randomized controlled trial; GEE, generalized estimating equations; HIV, human immunodeficiency virus; ICC, intra‐cluster correlation; ITT, intent‐to‐treat; PLHIV, People living with HIV; PN, peer navigation; SDI, same day initiation (of ART); SMS, short message service; TMLE, targeted maximum likelihood estimation; UTT, Universal Test & Treat.

## Supporting information

**Table S1.** Sensitivity analysis showing odds ratio estimates for retention in care derived from Targeted Maximum Likelihood Estimation
