# Supplementary material for: Impact of SMS and peer navigation on retention in HIV care among adults in South Africa: results of a three‐arm cluster randomized controlled trial
Source: J Int AIDS Soc. 2021 Aug 25;24(8):e25774. doi: 10.1002/jia2.25774 (PMC8387209; doi:10.1002/jia2.25774)
Supplement: Supplementary file 1 — Table S1. Sensitivity analysis showing odds ratio estimates for retention in care derived from Targeted Maximum Likelihood Estimation [file JIA2-24-e25774-s001.docx]

**APPENDIX**

**Table S1: Sensitivity analysis showing odds ratio estimates for retention in care derived from Targeted Maximum Likelihood Estimation**

|  | **Trial** |  |  | **Number** | **Percent** |  | **Odds** |  |  | |  |
| --- | --- | --- | --- | --- | --- | --- | --- | --- | --- | --- | --- |
|  | **Arm** | **N** |  | **Retained** | **Retained** |  | **Ratio** | **95% CI** | | | |
| **All Participants** | SOC | 167 |  | 63 | 37.72 |  | 1.00 |  | |  | |
|  | SMS-only | 289 |  | 116 | 40.14 |  | 1.15 | 0.46 | | 2.84 | |
|  | SMS+PN | 296 |  | 161 | 54.39 |  | 1.82 | 0.70 | | 4.74 | |
| **Males only** | SOC | 70 |  | 25 | 35.71 |  | 1.00 |  | |  | |
|  | SMS-only | 116 |  | 39 | 33.62 |  | 0.92 | 0.21 | | 3.99 | |
|  | SMS+PN | 106 |  | 53 | 50.00 |  | 1.61 | 0.60 | | 4.33 | |
| **Females only** | SOC | 97 |  | 38 | 39.18 |  | 1.00 |  | |  | |
|  | SMS-only | 173 |  | 77 | 44.51 |  | 1.28 | 0.46 | | 3.56 | |
|  | SMS+PN | 190 |  | 108 | 56.84 |  | 1.76 | 0.66 | | 4.69 | |
| **Pre-ART only** | SOC | 28 |  | 0 | 0.00 |  | *** |  | |  | |
|  | SMS-only | 55 |  | 2 | 3.64 |  | *** |  | |  | |
|  | SMS+PN | 41 |  | 5 | 12.20 |  | *** |  | |  | |
| **On ART only** | SOC | 139 |  | 63 | 45.32 |  | 1.00 |  | |  | |
|  | SMS-only | 234 |  | 114 | 48.72 |  | 1.15 | 0.58 | | 2.30 | |
|  | SMS+PN | 255 |  | 156 | 61.18 |  | 1.82 | 0.60 | | 5.53 | |
| **Pre-ART males only** | SOC | 14 |  | 0 | 0.00 |  | *** |  | |  | |
|  | SMS-only | 25 |  | 0 | 0.00 |  | *** |  | |  | |
|  | SMS+PN | 18 |  | 1 | 5.56 |  | *** |  | |  | |
| **Pre-ART females only** | SOC | 14 |  | 0 | 0.00 |  | *** |  | |  | |
|  | SMS-only | 30 |  | 2 | 6.67 |  | *** |  | |  | |
|  | SMS+PN | 23 |  | 4 | 17.39 |  | *** |  | |  | |
| **On ART males only** | SOC | 56 |  | 25 | 44.64 |  | 1.00 |  | |  | |
|  | SMS-only | 91 |  | 39 | 42.86 |  | 0.96 | 0.29 | | 3.18 | |
|  | SMS+PN | 88 |  | 52 | 59.09 |  | 1.57 | 0.65 | | 3.79 | |
| **On ART females only** | SOC | 83 |  | 38 | 45.78 |  | 1.00 |  | |  | |
|  | SMS-only | 143 |  | 75 | 52.45 |  | 1.34 | 0.29 | | 6.07 | |
|  | SMS+PN | 167 |  | 104 | 62.28 |  | 1.82 | 0.60 | | 5.57 | |

________________________________________________________________________________________

The causal inference models derived from Targeted Maximum Likelihood Estimation (TMLE) account for differences by clinic, age, marital status and travel time to nearest clinic. SOC = Standard of Care. SMS-only = Intervention using only short message service (text messaging). SMS+PN = intervention using SMS and peer navigation. ART = antiretroviral therapy. ***Note: there were no pre-ART participants retained in the SOC arm and a total of seven retained in the intervention arms. As such, in stratified analyses, we were not able to calculate odds ratios to compare outcomes between SOC and intervention arms for pre-ART participants.
